# Lipoma Arborescens of the Knee Joint

**Published:** 1908-03

**Authors:** Roland O. Meisenbach

**Affiliations:** Buffalo, N. Y.; Clinical Instructor in Orthopedic Surgery, University of Buffalo


### Lipoma Arborescens of the Knee Joint

By ROLAND O. MEISENBACH, M. D., Buffalo, N. Y.
Clinical Instructor in Orthopedic Surgery, University of Buffalo.

LIPOMA arborescens of the knee joint is a chronic disease, manifested by an overgrowth of lipomatous tissue on the synovial membrane. It may also exist in a lesser degree in the tendon sheaths near the joint and when occurring in the latter, gives rise to somewhat milder subjective symptoms than when occurring in the joint proper.

*Clinical Pathology.*—On examination of the knee it will be found that its entire surface is large and that the anatomical landmarks are usually obliterated. Both of the quadriceps pouches are enlarged and, upon palpation, a boggy mass may be felt; this is also frequently noted on the internal and external borders of the patella. Palpation elicits no pain or tenderness. Joint motion may or may not be restricted, this depending upon the location and severity of the growths.

*Gross Pathology.*—The gross pathology usually shows that the capsule and synovial membrane are overspread by masses of fusiform lipomata which project into the joint. These may occur singly or in clumps and may invest the entire joint cavity. The membrane itself is generally found to be in a fatty state, hanging loosely into the joint. It is sometimes difficult to distinguish the capsule from the surrounding soft parts, all being in a state of fatty degeneration which yields readily to the knife.

The lipomata hang loosely within the joint and are often seen to be attached to the articulations of the femur or tibia and even to have worked their way and attached themselves to the crucial ligaments. When this is the case, the lining membrane is continually dragged into the interarticular spaces whence subjective symptoms are noted. Upon active motion these adhesions are frequently broken up only to have new ones form at the same or another place. The upper border of the articular cartilage of the femur is a frequent place for these adhesions to form and, when found here, the capsule is anchored and its action limited.

Lipoma arborescens may be complicated by other chronic conditions of the joint such as osteo-arthritis, in which there may be spicules of bone or cartilage projecting from the articular surface, thus giving new points of attachment for the projecting growth. The latter condition was noted in one of the recent cases operated upon by me.

*Micropathology.*—Micropathology shows that the synovial structures are infiltrated by a mass of fatty globules; that fatty degeneration of the normal capsule exists and that, in the parts of the capsule surrounding these outgrowths of lipomata, there is a scarcity of bloodvessels, the adventitia of which also show fatty changes.

*Diagnosis.*—Lipoma arborescence of the knee joint usually occurs in patients who have taken on considerable weight. In women it is often noted about the age at which one would expect the menopause and may be associated with fatty degeneration of the heart and other organs. When occurring in individuals who have increased their weight within a comparatively short time, it exists generally as a local process, affecting one or both knees. When both knees are affected, the subjective symptoms may be much more pronounced in one than in the other; when this is the case the existing conditions in the knee least affected may easily be overlooked, but by closely questioning the patient, it will be found that at some time or another, the other knee has given trouble.

In the early stage and in the milder form, the patient complains of a dull ache in the knees without any definite localisation of the ache, but complains that the "cords" in the back of the knee (the hamstrings tendons and sheaths) are tired. Walking up stairs gives rise to slight pain at different times but this may not be constant. The pains may be referred or local, either of which may be severe and persist for a number of weeks and often disappear entirely for many months only to exacerbate at some future time in a severer form.

In this early stage of the affliction, the case may easily be mistaken for some of the other chronic conditions which, although they may be present, are not the causative factors of the symptoms. such as the so-called rheumatism and gouty diseases. It will be noted that, after urging the salicylates for over a long period, the symptoms still exist and no permanent relief has been afforded.

In the severer forms, very acute symptoms may be noted, such as sharp nauseating pains coming from a sudden motion, whence the case may simulate a loose semilunar cartilage or an acute attack of villous arthritis and even a hysterical knee. As in these, an acute exacerbation of the chronic condition may occur but, in lipoma arborescens, the pain is not so severe, allowing some use of the joint. The attacks may come on by a sudden mis-step or any sudden wrench of the knee joint or when a sitting posture is assumed quickly; that is, any motion in which the joint is suddenly thrown open.

The *x*-ray assists in making a diagnosis, more by eliminating than by confirming, although in a few cases the *x*-ray negative showed the fusiform lipomata very distinctly and it is regretted that they cannot be shown at this time. In order to show soft structures the plates must be so delicate in detail that they do not admit of reproducing in half tone but the lipoma must be demonstrated from the plates themselves.

*Treatment.*—This consists, essentially, in removing the cause by operative procedure although, by long and tedious immobilisation, the condition can sometimes be helped. The operative and post-operative technic must be well understood. The strictest asepsis must naturally be observed and the danger of entering a lipomatous knee joint must not be overlooked.

I herewith append a few cases of this nature.

Case I.—A brewer, 53 years of age, sent to me in April, 1907, a heavy built man, who walked with cane. He had always been well until six years ago when he noticed "rheumatism" settling in his right knee. At the onset there was a dull ache in the knee, but this gradually grew into a sharp pain after some months. He was entirely free from all subjective symptoms for weeks at a time but when they recurred, they seemed to return in a severer form. Upon advice, he tried anti-rheumatics, both internally and locally, off and on for some years but received no permanent relief from them. Plaster casts had also been worn with the same result. The knee had gradually been growing larger, the pains, upon walking, becoming so severe that for the last six months the patient was unable to attend to his work and had to use a cane. The physical examination of the knee showed the surface temperature to be normal; both quadriceps pouches and the lower anterior portion of the knee bulging. Knee held in a few degrees of flexion by spasm of the hamstrings. Palpation revealed no fluid in the joint, patella movable; the fulness in the quadriceps pouches and anterior portion suggested no fluid but rather a spongy mass within the joint, such as is felt in a Charcot joint. No crepitation on passive motion. *X*-ray in lateral position revealed a light shadow in the anterior portion of the knee joint, about the size of a ten cent piece. This shadow was constant after three plates were taken. The bones and cartilage of the joint were normal.

*Operation.*—Under ether, incisions, about ten cm. into the joint, were made on the inner and outer border of the patella. Considerable adipose tissue was penetrated before the joint was reached. Inspection showed the synovial membrane to be of a light yellow color and could not be easily distinguished from the surrounding tissue.

Exploration by the finger revealed small tufts of projecting masses over the entire synovial membrane. These tufts were soft and could be removed by the finger, except for a mass in the anterior portion near the alar ligament, which hung into the joint by a pedicle. This was removed by the scissors. After the synovial membrane had been thoroughly freed from all projecting tabs, the incisions were closed.

Three weeks after the operation, the patient could walk without a cane and had right angle flexion of the knee, although some fluid existed in the joint at this time. I did not see the knee again until eight months later, although I received letters from the patient saying that his knee gave him no trouble. On examination at this time, I found the knee normal, with normal motion.

CASE II.—A woman, 40 years old, weight 180 pounds, referred to me in April, 1906, gave the following history: when a girl she was of average weight but grew stout after marriage. Ten years ago, she wrenched her right knee and ever since, she has had sharp pains with local tenderness, chiefly noted while taking exercise or going up stairs. At that time, she consulted a surgeon who advised a leather splint which she wore at intervals. For the last few years she has had some aches in the small of her back. These were dull in character and were felt after standing for a long time. During the last year, her knee would suddenly go from under her and a sharp pain would be felt in the outer part of her right knee, "as if something slipped." These attacks became more freuqent, so that within the last few months, she has felt unsafe on her feet for fear of a sudden weakness of the knee joint.

Physical examination showed a large woman, very obese, with heavy hips and pendulous abdomen, unusually large and short thighs, with a marked bi-lateral knock-knee. The knee was so fat that the anatomical landmarks were entirely obliterated and the patella could scarcely be felt, being covered by about four cm. of firm adipose tissue. The anterior-posterior *x*-ray showed the joint to be in a state of chronic strain. The lateral view showed irregular shadows throughout the joint. A diagnosis of joint strain was made at the time, with a question of a loose body in the joint, and an operation was advised.

*Operation.*—The joint was entered on the internal aspect and explored with the finger. On the external side, near the outer condyle of the femur, a movable body was felt to be lodged in a pocket. On account of the depth of the tissue another incision was made on the outer side of the joint, parallel to the first, and through this a body about three cm. square was removed. Careful examination of the body made it evident that at some previous time it had been attached to the soft parts of the joint and that the continual churning between the articulation had torn it from its pedicle.

The joint was further explored and found to contain many fatty adhesions. The external quadriceps pouch was entirely bound down. Lipomata were seen projecting from all parts of the unattached lining membrane; these were removed and the incision was closed. The convalescence was uneventful. Owing to the existing joint strain, the patient was placed upon plates to prevent any undue strain to the knee. Two years have now elapsed without the slightest return of her former symptoms. The last report received was that she was playing tennis.

Case III.—A married woman, 39 years of age, a good liver, very stout, weighs about 160 pounds. Has always been well with the exception of eleven years ago, when she turned her right ankle and wrenched her right knee. Since that time, periodically once or twice a year, she has suffered a sudden wrench of the right knee joint,—the last attack occurring four months ago, which was caused by kicking back the train of her skirt with her right foot. Her knee suddenly gave way, she could not walk, and she fell to the floor.

Physical examination of the right knee showed an obesity of the entire structures with a slight rise in surface temperature. Slight ecchymosis noted in region of tubercle of the tibia. No fluid in joint but the entire joint area seemed boggy and the capsule clearly outlined. No tenderness over internal aspect of the knee. *X*-ray showed nothing of importance. This case was treated by the conservative method, first immobilising the joint, and later by a mechanical device. Operation had been suggested but refused, so there was nothing left to do but to give temporary relief. These attacks will undoubtedly return in the future.

Case IV.—A woman, 50 years old, single, always well until five years ago, when she contracted rheumatism which affected her knees and back. Operation for gallstones three years ago. Also has a right hernia which is held in place by a bandage. Patient has taken on much weight in the last six years. The pain in the knees was usually of a dull character but at times she would feel a sudden catch in the right knee, which caused her nauseating pain. Gradually she noticed that her knees were growing stiffer and that going up and down stairs gave her constant pain. Has had several acute attacks of pain in the knees which were brought on by taking a high step, such as getting on a car. After resting the knees for a few days, the pain would become less. The last attack occurred six weeks ago, when she was referred to me.

*Physical examination.*—Well developed and nourished woman, obese, with large flabby abdomen. Cardiac lesion present, skin dry. Knees.—*Left*: surface temperature normal, no crepitation on motion, upper quadriceps pouches somewhat full, patella tendon firm, no tenderness on palpation. *Right*: some fullness in external quadriceps pouch, surface temperature slightly plus, hamstrings tense and in state of spasm, knee held in flexed position. Active and passive motion elicits pain and slight crepitation. Some tenderness in region of the ham strings. *X*-rays of the right knee show multitudes of small shadows in region of capsular portion of the joint and in region of the hamstrings tendons, the latter being distinctly visible on the plate. The periarticular structures show a mottled appearance suggesting lipomatous degeneration of tissue. The patella shows small spicules of bone projecting from its articular surface. The edges of the condyle appear rough, also present small projecting spicules. Diagnosis: lipoma arborescens and osteo-arthritis.

*Operation.*—Two incisions were made parallel to the patella directly down to the joint. Through the outer incision, the lining membrane of the joint, plainly visible, hung in loose folds into the articular space. From these folds emanated numerous lipomata, some singly, others grouped together. The capsule was bound down by these lipomata which were adherent to the femur, some of which overlapped the articular cartilage. On the external condyle was noted an exostosis around which were caught folds of lipomatous membrane. Hence, at each motion of the joint, considerable tension must have taken place and a continual irritation kept up. On the articular surface of the patella, close to the edge, spicules of ostoid tissue were found.

The operation consisted, essentially, in breaking up the lipomatous adhesions with the finger and removing the projecting lipomata with the scissors. The exostoses were removed with the osteotome. After flushing the joint, the incisions were closed. Three weeks after the operation, the patient was able to walk and climb stairs without limitation to motion and without recurrence of the former symptoms.

140 Allen Street.

---

"Yes, doctor, one of Harry's eyes seems ever so much stronger than the other. How do you account for that?"

"Knothole in the baseball fence last summer, most likely, madam."—*Harper's Weekly.*

